# ﻿Revised diagnoses of the gudgeons *Belobranchusbelobranchus* and *B.segura* (Actinopterygii, Gobiiformes, Eleotridae)

**DOI:** 10.3897/zookeys.1232.141880

**Published:** 2025-03-17

**Authors:** Muhammad Afrisal, Nurjirana Nurjirana, Haryono Haryono, Daniel Frikli Mokodongan, Kunto Wibowo, Abigail Mary Moore, Nicolas Hubert

**Affiliations:** 1 Museum Zoologicum Bogoriense, Research Center for Biosystematics and Evolution, National Research and Innovation Agency, Cibinong 16911, Indonesia National Research and Innovation Agency Cibinong Indonesia; 2 Capture Fisheries Department, Indonesia Defense University, Belu 85752, Indonesia Indonesia Defense University Belu Indonesia; 3 Graduate School, Hasanuddin University, Makassar 90245, Indonesia Hasanuddin University Makassar Indonesia; 4 ISEM, Université de Montpellier, CNRS, IRD, Montpellier, France Université de Montpellier Montpellier France

**Keywords:** COI, coloration, distribution, Indonesia, morphology, morphometrics

## Abstract

Diagnostic characters distinguishing the gudgeons *Belobranchusbelobranchus* and *Belobranchussegura* were reassessed and found to be inconsistent, making it difficult to accurately identify them. Numerous specimens of both species were examined combining genetic (mitochondrial COI gene) and morpho-meristic analyses. Our findings demonstrate that *B.belobranchus* and *B.segura* can be reliably distinguished from each other based on revised diagnostic characters, including several morphometric features (interorbital width, jaw length, and caudal-peduncle depth) as well as distinct head, body, and fin coloration.

## ﻿Introduction

The eleotrid genus *Belobranchus* Bleeker, 1856 is distinguishable from all other genera in the family Eleotridae by a unique combination of morphological characteristics (Kottelat et al. 1993; Larson and Murdy 2001). Members of this genus have a scaleless head covered with sensory papillae arranged in a longitudinal pattern. The head lacks elongated papillae or branched barbels, and there is no distinct ridge above the eye or on the dorsal surface of the snout. Additionally, the first branchiostegal ray, or sometimes the first and second rays, bears an anteriorly directed spine. The genus comprises two species, *Belobranchusbelobranchus* (Valenciennes, 1837) and *Belobranchussegura* Keith, Hadiaty & Lord, 2012, which have overlapping distributions in the eastern Indian Ocean and western Pacific. *Belobranchusbelobranchus* was originally described as *Eleotrisbelobrancha* based on five syntypes from Manado Island, Sulawesi, Indonesia, while the latter species was described from 13 specimens from several rivers of West Papua and Halmahera, Indonesia.

In the original description of *B.segura*, Keith et al. (2012) distinguished the new species from *B.belobranchus* based on meristic counts (number of scales in the transverse forward, transverse back, and pre-dorsal series) and coloration of the body and fins (including dorsal, anal, pectoral, and caudal fins). However, molecular and morphological examinations of both type and numerous non-type specimens of *B.segura* and *B.belobranchus* in this study revealed variability in most of the diagnostic characters provided by Keith et al. (2012).

Although this genus consists of only two species, the unclear diagnostic characters make it difficult to accurately identify them. This study provides a revised morphological diagnosis, including morphometric characters and coloration patterns of the head and body, to precisely distinguish the two species.

## ﻿Material and methods

All counts and measurements were taken from the left side of the body, unless the left side was damaged. Counts and measurements generally followed Watson (1995) and Nakabo (2002), with the following modifications: head width and head depth were measured at the posterior margin of the preopercle, snout width was defined as the distance between the nostrils, and body width was measured at the origin of the pectoral fin. Measurements were taken point-to-point with digital calipers to the nearest 0.01 mm. Meristic counts were determined through observation under a stereomicroscope (Nikon SMZ460). Standard length is abbreviated as SL. Sex was determined by confirming the shape of the urogenital papilla (in males, the urogenital papilla long or triangular with pointed distal tip, whereas in females, the urogenital papilla somewhat bulbous in appearance with a fimbriate distal tip). Observations of fresh coloration were based on photographs deposited on the Barcode of Life Data System website (BOLD, www.barcodinglife.org) and the newly collected specimens in this study. All specimens of the genus *Belobranchus* examined in this study are deposited at the Museum Zoologicum Bogoriense, Indonesia (**MZB**; Bogor).

Phylogenetic analysis was performed in MEGA 11 (Tamura et al. 2021) to visualize the evolutionary divergence between *Belobranchus* species, and the final tree was edited in the Inkscape 1.2.2. The 38 sequences of the cytochrome c oxidase I (COI) mitochondrial gene of the species of *Belobranchus*, accession numbers KU692344.1–KU692375.1 and KU692379.1 (Dahruddin et al. 2016); MT706791.1, MT706722.1, MT706726.1, MN045250.1, MN069306.1, and MN069308.1 (Sahami and Habibie 2021), and a sequence of *Eleotrisfusca*MN045250.1 (Mennesson et al. 2019), used for phylogenetic reconstruction in this study are currently available in the NCBI GenBank database (www.ncbi.nlm.nih.gov/). Additionally, a sequence of *Eleotrisfusca* (Bloch & Schneider, 1801) (KU692479.1) was included as an outgroup. The sequences were aligned using CLUSTALW 1.6 (Higgins et al. 1996) and trimmed to produce 652 bp homologous fragments of the COI gene. Phylogenetic relationships among sequences were inferred using the Neighbor-Joining algorithm (Saitou and Nei 1987) based on the Kimura 2-Parameter (K2P) model (Kimura 1980). Genetic distances (number of base substitutions per site) were computed using the Maximum Composite Likelihood method (Tamura et al. 2004) with 1,000 bootstrap replications (Felsenstein 1985). Graphs of morphometric relationships were generated using Microsoft Excel.

## ﻿Results

### ﻿ Belobranchus belobranchus

Taxon classificationAnimaliaGobiiformesEleotridae

(Valenciennes, 1837)

BD242A8D-21B8-5751-AEAB-81C2289F235E

**Figure 1.**
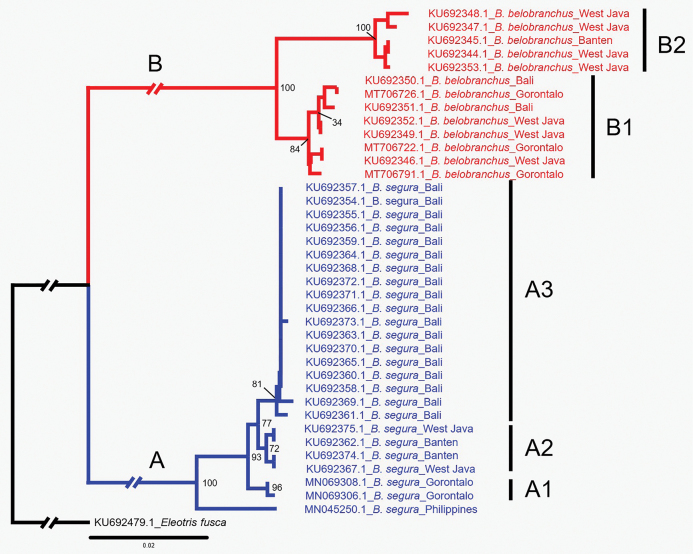
Neighbor-join phylogenetic tree of COI GenBank accessions for the genus *Belobranchus* [Kimura 2-Parameter model (K2P); 1000 bootstrap replications; *Eleotrisfusca* as outgroup].

**Figure 2.**
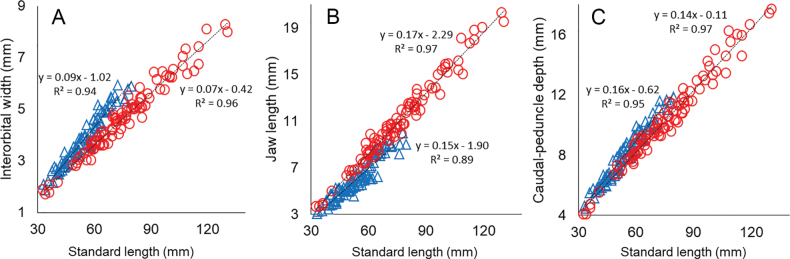
Morphometric relationships in *Belobranchusbelobranchus* (circles) and *B.segura* (triangles) **A** interorbital width **B** jaw length **C** caudal-peduncle depth.

**Figure 3.**
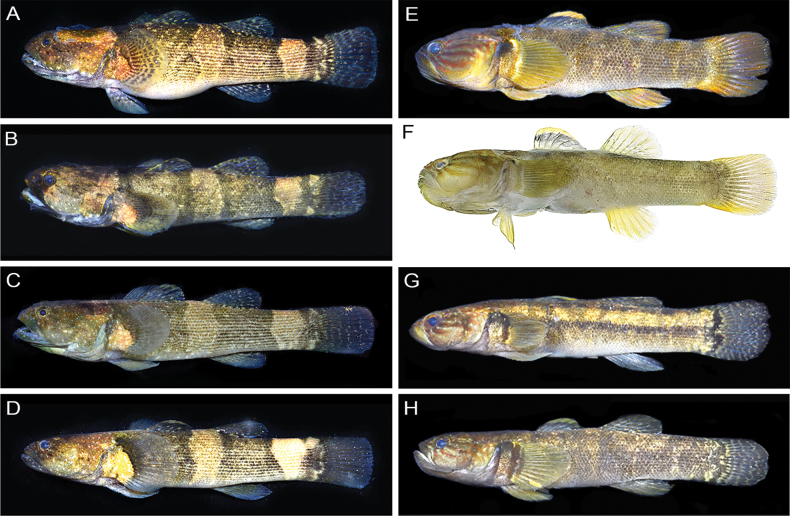
Fresh specimens of *Belobranchus* species **A–D***B.belobranchus***A**MZB.26873, 77.5 mm SL **B**MZB.26907, 74.8 mm SL **C**MZB.26872, 71.7 mm SL **D**MZB.26906, 58.7 mm SL) **E–H***B.segura***E**MZB.26902, 68.9 mm SL **F**MZB.26687, 59.6 mm SL **G**MZB.26668, 56.3 mm SL **H**MZB.26894, 52.6 mm SL.

Figs 1 , 2 , 3A–D , 5A , 6 , Tables 1 , 2 English name: Throat-spine Gudgeon

Eleotris belobrancha Valenciennes in Cuvier and Valenciennes 1837: 243 (type locality: Manado Island, Sulawesi, Indonesia)
Belobranchus quoyi Bleeker, 1856: 300 (type locality: Manado Island, Sulawesi, Indonesia)
Belobranchus taeniopterus Bleeker, 1856: 301 (type locality: Boleling, Bali, Indonesia)

#### Material examined.

***Non-type specimens*.** 94 specimens, all specimens from Indonesia. **Java**: • MZB.16307, 84.6 mm SL, male, Cibareno, Pasir Baru, Cisolok, Sukabumi, Jawa Barat, S. Sauri et al., 23 Jun. 2002 • MZB.16328, 63.1 mm SL, female, Cilumajan, Pasir Baru, Cisolok, Sukabumi, Jawa Barat, S. Sauri et al., 24 May 2002 • MZB.26872 [ex BIF.01651], 71.7 mm SL, male, Cibareno, Lebak, Banten, 6°57'48.0"S, 105°23'42.0"E, N. Hubert et al., 10 Dec. 2013 • MZB.26873 [ex BIF.1696], 77.5, female, MZB.26906 [ex BIF01694], 58.7 mm SL, male, Citiis, Sukabumi, Jawa Barat, 6°56'46.0"S, 106°26'45.6"E, N. Hubert et al., 11 Dec. 2013 • MZB.23934, 33.9 mm SL, male, Purworejo, Jawa Tengah, R. Hadiaty, 10 Sep. 2017. **Bali**: • MZB.26874 [ex BIF.2385], 74.8 mm SL, male, MZB.26907 [ex BIF.2384], 65.0 mm SL, female, Nbang, Jembrana, Bali, 8°22'01.2"S, 114°45'07.2"E, N. Hubert et al., 15 Apr. 2014. **Sulawesi**: • MZB.4795, 67.4–106.9 mm SL, 2 specimens, male and female, Watu Songu, Ulubongka, Poso, Sulawesi Tengah, Suyanto, 10 Nov. 1982 • MZB.11665, 70.1–108.6 mm SL, 3, 1 male, 2 females, MZB.11676, Lambuno, Wana Mukti, Mountong, Donggala, Sulawesi Tengah, Agus, 3 May 2002 • MZB.11715, 45.4–52.1 mm SL, 6 specimens, 2 males, 4 females, Haryono and A. Munim, 8 Aug. 2001 • MZB.11731, 66.1–100.2 mm SL, 8 specimens, 2 males, 6 females, Wapo river, Lombongo, Suwawa, Gorontalo, Haryono and A. Munim, 7 Aug. 2001 • MZB.11735, 57.3–65.2 mm SL, 4 specimens, 3 males, 1 female, Tolumolu, Kota Selatan, Sulawesi Utara, 8 Aug. 2001 • MZB.11964, 109.5 mm SL, female, MZB.11975, 113.0 mm SL, female, MZB.12048, 62.3 mm SL, female, Bolaang Mongondow, Sulawesi Utara, Haryono and Hesron, 21–26 May 2002 • MZB.20004, 58.6–77.1 mm SL, 3 specimens, 1 male, 2 females, Masembo, Mekongga, Tinukari, Wawo, Kolaka Utara, Sulawesi Tenggara, Jumaring, 30 Nov. 2010 • MZB.20352, 36.4–115.4 mm SL, 17 specimens, 11 males, 6 females, MZB.20381, 52.9–96.2 mm SL, 9 specimens, 5 males, 4 females, MZB.20391, 78.9 mm SL, female, MZB.20398, 107.0–129.5 mm SL, 3 specimens, 2 males, 1 female, MZB.20406, 58.5–63.8 mm SL, 3 specimens, 1 male, 2 females, MZB.20415, 51.0–100.3 mm SL, 3 males, Tinukari, Wawo, Kolaka Utara, Sulawesi Tenggara, R. Hadiaty et al., 28 Jun.–3 Jul. 2011 • MZB.22703, 84.1 mm SL, male, Bonepuso, Bulagi Selatan, Banggai Kepulauan, S. Sauri, 20 Sep. 2014 • MZB.24579, 101.4 mm SL, male, Marinding, Luwu, Sulawesi Selatan, 3°21'43.5"S, 120°15'54.3"E, seine net, D. Lumbantobing, 12 Aug. 2016. **Alor**: • MZB.5346, 115.3 mm SL, male, Alor, Nusa Tenggara Timur, Sudarmanu, Apr. 1984. **Halmahera**: • MZB.18812, 85.3 mm SL, male, MZB.18830, 61.3–79.7 mm SL, 3 males, MZB.21174, 32.8 mm SL, male, MZB.21184, 93.4 mm SL, female, MZB.21209, 71.8–98.4 mm SL, 5 specimens, 1 male, 4 females, MZB.21219, 58.2 mm SL, female, MZB.26661 [ex MZB.18747], 63.3–130.7 mm SL, 7 specimens, 1 male, 6 females, MZB.26662 [ex MZB.18820], 71.8 mm SL, female, Ake Jira, Weda Tengah, R. Hadiaty et al., 4 Feb. 2010–16 Jul. 2011. **Papua**: • MZB.15631, 95.2 mm SL, female, Waigeo, Raja Ampat, Papua, R. Hadiaty et al., 4 Jun. 2007 • MZB.17098, 65.3–97.6 mm SL, 2 males, MZB.17118, 59.7–61.0 mm SL, male and female, MZB.17140, 91.9 mm SL, male, Batanta, Raja Ampat, Papua Barat, R. Hadiaty et al., 30 July–5 May 2008.

**Table 1.** Counts and measurements (expressed as percentages of standard length) of *B.belobranchus* and *B.segura*.

	*B.belobranchus*		*B.segura*			
	Non-type specimens		Holotype	Paratypes	Non-type specimens	
	n = 94		MZB.20786	n = 9	n = 78	
Standard length (SL, mm)	32.8–130.7	mode	72.1	41.0–72.5	33.4–79.8	mode
**Counts**						
Pored lateral-line scales	51–62	56 or 57	59	53–58	50–62	54 or 56
Caudal-fin rays	13	13	13	13	13	13
Pectoral-fin rays	20–23	20	21	20–22	20–22	20
Transverse backward scales	17–23	19	19	19–21	16–21	19
Transverse forward scales	18–34	22	26	22–27	18–28	22
Pre-dorsal scales	20–39	26	20	16–23	16–32	24
Zigzag series	17–21	19	19	17–19	17–21	19
**Measurements (% SL)**		**mean**				**mean**
Head length	26.6–33.1	30.4	29.5	28.3–31.4	23.9–30.9	28.6
Head width	17.6–29.2	24.7	23.3	20.2–24.6	16.8–26.4	21.8
Head depth	12.1–20.0	16.5	16.7	13.9–18.3	13.0–19.8	16.0
Mouth width	8.7–21.7	16.1	13.0	9.7–15.0	8.5–16.4	12.0
Eye diameter	3.7–5.9	4.9	6.2	5.5–7.0	4.4–7.0	5.7
Snout width	4.9–7.1	6.1	7.4	6.2–8.2	5.6–8.4	7.0
Interorbital width	4.8–8.0	6.0	11.6	7.4–11.0	6.4–11.2	8.2
Distance snout to isthmus	9.1–14.6	11.2	10.8	10.5–12.9	8.7–15.2	11.7
Postocular length	13.7–21.3	17.4	16.2	13.3–17.7	12.6–17.5	15.6
Jaw length	10.3–16.7	14.1	12.3	10.1–12.9	8.7–13.5	11.1
Body depth	15.2–21.8	18.6	21.1	18.3–22.0	15.7–20.9	18.9
Body width	10.7–19.9	15.5	16.2	13.6–17.5	10.5–17.9	14.5
Pre-dorsal-fin length	38.3–44.8	41.5	41.1	38.1–41.5	36.8–42.6	40.2
Snout to second dorsal-fin origin length	55.6–65.6	60.6	61.9	59.4–62.3	54.8–63.1	59.7
Second dorsal-fin length	18.8–23.8	21.6	25.0	21.3–24.1	19.8–25.7	22.9
Length of first dorsal-fin base	9.8–14.1	11.9	12.9	11.7–14.8	10.4–15.6	12.8
Length of second dorsal-fin base	10.2–13.3	11.3	11.4	10.1–12.1	10.3–13.5	11.6
First dorsal-fin origin to second dorsal-fin origin	17.2–22.8	19.6	21.2	19.8–22.6	17.7–22.9	20.2
First dorsal-fin origin to pelvic-fin origin	15.8–24.8	21.3	22.3	18.7–22.2	16.0–23.5	20.4
First dorsal-fin origin to anal-fin origin	25.7–32.0	29.0	31.2	28.1–30.4	25.8–31.9	29.1
Second dorsal-fin origin to anal-fin origin	14.2–21.0	18.1	20.8	16.6–20.7	15.8–20.7	18.2
Interval between first and second dorsal-fin bases	5.2–9.8	7.7	8.5	7.4–9.3	4.2–10.3	7.3
Pelvic-fin origin to anal-fin origin	30.0–41.2	34.8	35.5	33.6–35.3	29.1–38.8	33.9
Pelvic-fin origin to second dorsal-fin origin	31.4–40.9	36.2	37.3	33.0–37.7	32.2–39.7	36.1
Anal-fin length	18.5–23.5	21.4	23.7	21.4–23.5	20.1–24.1	22.2
Pre-anal-fin length	62.2–70.6	65.9	64.9	63.7–66.4	60.7–68.3	64.8
Length of anal-fin base	8.4–11.9	9.8	10.6	8.7–10.2	8.5–12.5	10.2
Caudal-peduncle depth	11.9–15.0	13.6	15.5	13.5–16.0	13.2–16.6	14.7
Caudal-peduncle length	19.6–29.0	22.4	27.2	26.0–27.6	20.0–31.7	24.4
Caudal-fin length	19.7–25.1	22.4	23.8	20.1–23.9	18.8–24.1	21.4

#### Diagnosis.

A species of *Belobranchus* with the following combination of characters: head relatively depressed; interorbital width 4.8–8.0% (mean 6.0%) of SL; jaw length 10.3–16.7% (14.1%) of SL; caudal-peduncle depth 11.9–15.0% (13.6%) of SL; body with 3 dark-brown bands seaparated by pale bands, with many dark horizontal lines (one per scale row) along lateral surface; dorsal and lateral surfaces of head with many small whitish spots; 1 or 2 distinct oblique brown lines extending from eye to posterior edge of operculum (not always visible); first dorsal fin typically uniformly mottled dark brown or with thin yellowish distal edge; largest recorded specimen 130.7 mm SL.

#### Distribution and habitat.

*Belobranchusbelobranchus* has been recorded from the South China Sea (Randall and Lim 2000), Indonesia: Java, Bali, Lombok, Flores, Alor, Timor, Buton, Sulawesi, and Halmahera islands, and West Papua (Roberts 1993; Tjakrawidjaja 2002; Larson and Pidgeon 2004; Larson et al. 2007; Keith et al. 2012; Tweedley et al. 2013; Dahruddin et al. 2016; Miesen et al. 2016; this study); the Philippines (Jamandre 2023); Japan (Sakai et al. 2001; Nakabo 2002); Papua New Guinea (Fricke et al. 2014; Amick and Toko 2021) (Fig. 6). *Belobranchusbelobranchus* is an amphidromous species, characterized by migration between freshwater and coastal environments (Keith et al. 2012; Sahami et al. 2019); its habitat predominantly encompasses the lower regions of rivers, typically at altitudes to 5 m, and it has been observed in muddy to clear river systems featuring sandy to rocky or gravel bottoms (Parenti 2021). Like most eleotrids, this fish is carnivorous (Kiruba-Sankar et al. 2018).

### ﻿ Belobranchus segura

Taxon classificationAnimaliaGobiiformesEleotridae

Keith, Hadiaty & Lord, 2012

534C89B2-0A95-5B0D-858A-50231C77CE62

**Figure 4.**
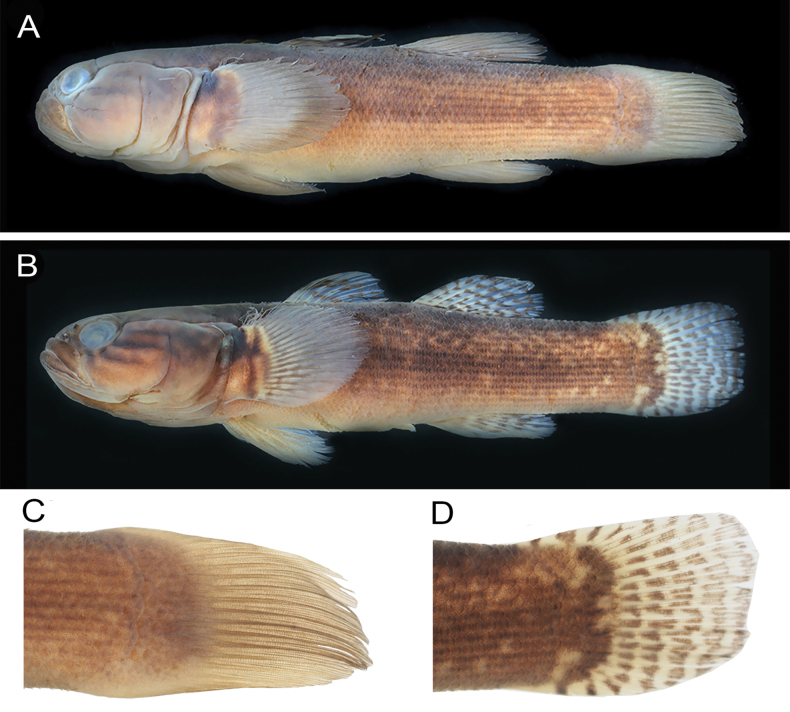
Preserved specimens of *B.segura***A**MZB.20786, holotype, 72.1 mm SL **B**MZB.18747, paratype, 55.9 mm SL **C, D** caudal fins of the holotype and the paratype, respectively.

**Figure 5.**
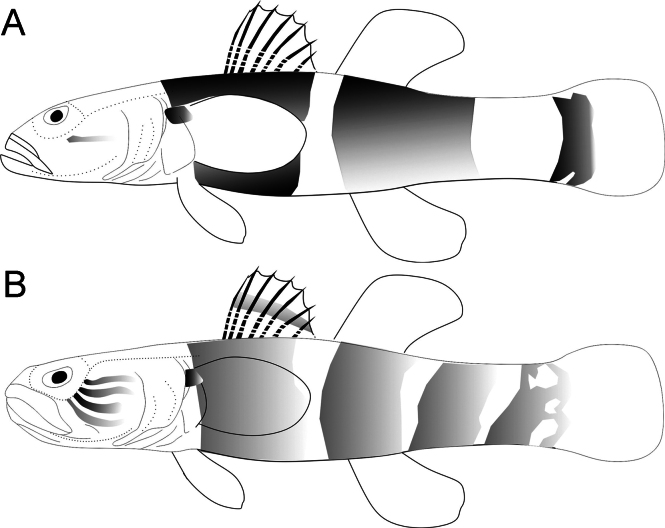
Schematic illustration of the number and patterns of lines on the cheek and lateral bands on the body of *Belobranchus* species **A***B.belobranchus***B***B.segura*.

**Figure 6.**
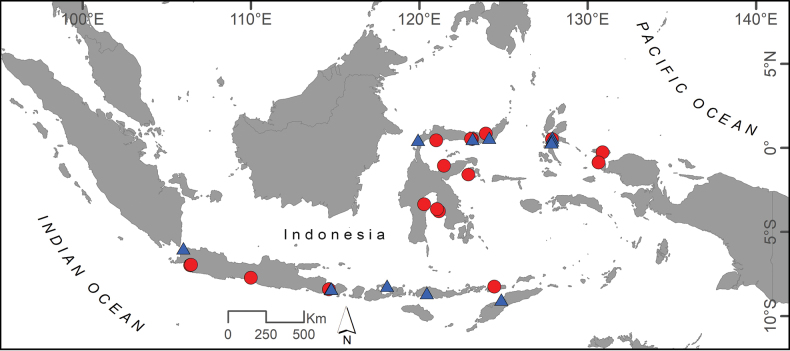
Distributional records of *B.belobranchus* (circles) and *B.segura* (triangles) based on collected specimens examined in this study.

Figs 1 , 2 , 3E–H , 4 , 5B , 6 , Tables 1 , 2 New English name: Segura Gudgeon

Belobranchus segura Keith, Hadiaty & Lord, 2012: 480, figs 1, 2, 3A, 3C (type locality: Ake Jira, Leililef Waibulen, Halmahera, Indonesia)

#### Material examined.

***Holotype*.**MZB.20786, 72.1 mm SL, male, Ake Jira, Leililef Waibulen, Halmahera, Indonesia, R. Hadiaty et al., 26 Jan. 2010.

***Paratypes*.** 9 specimens, 40–72 mm SL, all specimens collected from Maluku Island, Indonesia. • MZB.18658, 61–66 mm SL, 2 males, Ake Kobe, Halmahera, R. Hadiaty et al., 23 Jan. 2010 • MZB.18684, 64–72 mm SL, 3 males, same data as holotype • MZB.18747, 55 mm SL, female, MZB.18751, 40–44 mm SL, 2 females, MZB.18820, 44 mm SL, female, Ake Jira, Weda Tengah, Halmahera, R. Hadiaty et al., 4–7 Feb. 2010.

#### Non-type materials.

78 specimens, 33.4–79.8 mm SL, all specimens collected from Indonesia. **Java**: • MZB.23934, 33.9 mm SL, male, Purworejo, Jawa Tengah, R. Hadiaty, 10 Sep. 2017 • MZB.26667 (ex BIF.1483), 42.1 mm SL, male, MZB.26890 [ex BIF.1482], 39.1 mm SL, male, Cibeber, Pandeglang, Banten, 6°35'29.0"S, 105°37'48.0"E, N. Hubert et al., 7 Dec. 2013. **Bali**: • MZB.26668 (ex BIF.2389), 56.3 mm SL, female, MZB.26891 (ex BIF.2386), 50.2 mm SL, female, MZB.26892 (ex BIF.2374), 55.1 mm SL, male, MZB.26893 (ex BIF.2375), 61.5 mm SL, male, MZB.26894 (ex BIF.2387), 52.6 mm SL, female, MZB.26895 (ex BIF.2373), 59.0 mm SL, male, MZB.26896 (ex BIF.2376), 60.8 mm SL, male, MZB.26897 (ex BIF.2388), 35.9 mm SL, female, Nbang, Jembrana, Bali, 8°22'01.2"S, 114°45'07.2"E, N. Hubert et al., 15 Apr. 2014 • MZB.26670 (ex BIF.2460), 52.0 mm SL, female, MZB.26898 (ex BIF.2457), 60.3 mm SL, male, MZB.26899 (ex BIF.2456), 65.1 mm SL, male, MZB.26900 (ex BIF.2459), 55.3 mm SL, male, MZB.26901 (ex BIF.2454), 69.2 mm SL, male, MZB.26902 (ex BIF.2455), 68.9 mm SL, male, Yeh Sumbul, Jembrana, Bali, 8°21'36.0"S, 114°47'02.4"E, N. Hubert et al., 16 Apr. 2014. **Sulawesi**: • MZB.11715, 41.3–52.1 mm SL, 6 specimens, 2 males, 4 females, MZB.11717, 46.1–68.0 mm SL, 6 specimens, 3 males, 3 females, MZB.11727, 37.8–62.2 mm SL, 6 specimens, 4 males, 2 females, Baliohuto, Lombongo, Suwawa, Gorontalo, Haryono and A. Munim, 6–8 Aug. 2001 • MZB.12048, 62.3 mm SL, female, Molong, Pusian Dumoga, Bolaang Mongondow, Sulawesi Utara, Haryono and Hesron, 26 May 2002 • MZB.26663 (ex MZB.11676), 47.3–69.4 mm SL, 11 specimens, 6 males, 5 females, Mountong, Donggala, Sulawesi Tengah, Agus, 2 May 2002 • MZB.26664 (ex MZB.1197), 61.2–76.1 mm SL, male and female, Bolaang Mongondow, Sulawesi Utara, Haryono and Hesron, 22 May 2002 • MZB.26669 (ex MZB.22703), 49.1–50,4 mm SL, male and female, Bonepuso, Bulagi Selatan, Banggai Kepulauan, S. Sauri, 20 Sep 2014. **Lombok**: • MZB.22933, 42.0–50.5 mm SL, 3 females, Ormori, Kawinda Toi, Tambora, Sumbawa, Nusa Tenggara Barat, Mulyadi, 24 Apr. 2015. **Flores**: • MZB.6359, 43.4–53.5 mm SL, 4 specimens, 2 males, 2 females, Wae Laku, Ruteng, Flores, Nusa Tenggara Timur, Agus and Munir, 26 May 1994. **Timor**: • MZB.26903, 59.6 mm SL, male, MZB.26904, 59.6 mm SL, male, MZB.26905, 57.8 mm SL, male, MZB.26906, 44.4 mm SL, male, MZB.26908, 66.1 mm SL, male, Jenilu, Kakuluk Mesak, Belu, Nusa Tenggara Timur, 9°00'41.8"S, 124°52'52.2"E, M. Afrisal et al., 23 Jan. 2023. **Halmahera**: • MZB.18700, 67.2 mm SL, male, MZB.18706, 76.4–79.8 mm SL, 3 males, MZB.18711, 56.7 mm SL, female, MZB.26909 (ex MZB.18820), 59.0–58.1 mm SL, 2 females, Ake Jira, Weda Tengah, R. Hadiaty et al., 27 Jan.–7 Feb. 2010 • MZB.21219, 58.2 mm SL, female, Ake Kobe, Weda Tengah, R. Hadiaty et al., 7 Jul. 2011 • MZB.26665 (ex MZB.18747), 38.4–43.8 mm SL, 2 females, MZB.26666 (ex MZB.18751), 33.4–43.0 mm SL, 4 females, Ake Saki, Weda Tengah, R. Hadiaty et al., 7 Feb. 2010.

#### Diagnosis.

A species of *Belobranchus* with the following combination of characters: head somewhat convex; interorbital width 6.4–11.6% (mean 8.2%) of SL; jaw length 8.7–13.5% (11.1%) of SL; caudal-peduncle depth 13.2–16.6% (14.7%) of SL; body with 4 or 5 dark-brown bands separated by narrow pale bars, lacking dark horizontal lines; dorsal and lateral surfaces of head without small whitish spots; 3 or 4 oblique brown lines extending from eye to posterior edge of operculum; upper margin of first dorsal fin yellowish to orange; largest recorded specimen 79.8 mm SL.

#### Distribution and habitat.

*Belobranchussegura* has been recorded from Indonesia: Sumatra, Java, Bali, Lombok, Sulawesi, Halmahera, Sumbawa, Flores, Timor, Papua Barat (Kottelat et al. 1993; Keith et al. 2012; Dahruddin et al. 2016; Miesen et al. 2016; Sahami and Habibie 2021; Hasan et al. 2023; this study), the Philippines (Vedra et al. 2013), Japan (Suzuki et al. 1995), Papua New Guinea (Amick and Toko 2021: fig. 2P,Q), Solomon Islands and Vanuatu (Keith et al. 2012), and Philippines (Gonzalez et al. 2023) (Fig. 6). This species is considered amphidromous (Keith et al. 2012; Sahami and Habibie 2021) and has been found in the lower reaches of muddy to clear rivers with sandy to gravel substrate, to 5 m in altitude, close to estuaries. Like most eleotrids, this fish is considered carnivorous (Kiruba-Sankar et al. 2018).

## ﻿Discussion

The *Belobranchus* phylogenetic tree based on partial COI (652 bp) sequences reveals two main clades with a 14.4% mean pairwise genetic distance (Fig. 1). The monophyletic group of *B.segura* in Clade A includes one sequence from the Philippines (previously identified as *B.belobranchus*; see Mennesson et al. 2019) along with all other *B.segura* sequences, while clade B, containing all *B.belobranchus* sequences, also forms a monophyletic group. This clade is supported by a bootstrap proportion of 90%. Clade A corresponds to the geographic areas as follows: A1 from Tomini Bay, Gorontalo, Sulawesi; A2 from Java (West Java and Banten); and A3 from West Bali. Clade B is divided into two clusters, B1 containing sequences from various origins and B2 comprised entirely of sequences from West Java. The pairwise genetic distances among the *B.segura* clusters (A1, A2 and A3) were ca 1.0% on average, while the mean pairwise genetic distance was 3.4% between the two *B.belobranchus* clusters (B1 and B2). However, morphological examinations based on the diagnostic characters given by Keith et al. (2012), including meristic and color characters, did not correspond well with the results of the phylogenetic analysis.

In the paper by Keith et al. (2012), there were also inconsistencies in the stated number of specimens between the material examined and results section. The number of *B.belobranchus* specimens was stated as 13 in the material examined, but data were provided for 14 specimens in the results (see Keith et al. (2012): table 1: transverse backward series). There was a similar discrepancy for *B.segura*, with a total of 13 specimens described in the material examined, but data from 14 specimens included in the results.

The meristic data presented in Keith et al. (2012: table 1), including the transverse backward scale, transverse forward scale, and pre-dorsal scale counts, initially appeared sufficient to distinguish between *B.segura* and *B.belobranchus*. However, upon examining numerous specimens of both species, the range of values for each character expanded, resulting in significant overlap, suggesting that these characters may not be reliable for distinguishing between the two species (Table 2).

**Table 2.** Frequency distributions of selected counts of *B.belobranchus* and *B.segura*.

	n	Pored lateral–line scales															n	Transverse backward series							
		50	51	52	53	54	55	56	57	58	59	60	61	62	63			16	17	18	19	20	21	22	23
*B.belobranchus* (Keith et al. 2012)	13	–	–	–	–	–	–	1	1	3	2	3	1	1	1		14	–	–	–	–	4	4	4	2
*B.segura* (Keith et al. 2012)	13	–	–	–	–	–	2	3	1	2	2	3	–	–	–		14	–	–	3	6	4	1	–	–
*B.belobranchus* (this study)	94	1	–	–	2	13	9	16	16	16	13	7	2	1	–		94	–	2	3	53	24	11	2	1
*B.segura* (this study)	88	2	1	6	8	18	8	18	13	6	5	1	1	1	–		88	1	3	9	58	12	5	–	–
	**n**	**Transverse forward series**																							
		**18**	**19**	**20**	**21**	**22**	**23**	**24**	**25**	**26**	**27**	**28**	**29**	**30**	**31**	**32**	**33**	**34**	**35**						
*B.belobranchus* (Keith et al. 2012)	13	–	–	–	1	–	1	3	3	3	2	–	–	–	–	–	–	–	–						
*B.segura* (Keith et al. 2012)	13	–	–	–	–	–	–	–	–	–	2	2	2	2	2	–	1	1	1						
*B.belobranchus* (this study)	94	1	–	2	–	30	1	22	–	17	2	10	1	4	–	3	–	3	–						
*B.segura* (this study)	88	2	1	16	3	32	2	19	2	8	1	2	–	–	–	–	–	–	–						
	**n**	**Pre–dorsal midline series**																							
		**16**	**17**	**18**	**19**	**20**	**21**	**22**	**23**	**24**	**25**	**26**	**27**	**28**	**29**	**30**	**31**	**32**	**33**	**34**	**35**	**36**	**37**	**38**	**39**
*B.belobranchus* (Keith et al. 2012)	14	–	–	–	–	–	2	1	1	–	3	1	1	1	1	–	–	–	–	1	–	–	–	–	–
*B.segura* (Keith et al. 2012)	9	1	1	1	–	1	1	1	3	–	–	–	–	–	–	–	–	–	–	–	–	–	–	–	–
*B.belobranchus* (this study)	94	–	–	–	–	4	1	5	2	7	6	14	7	8	13	6	3	8	3	2	1	2	–	1	3
*B.segura* (this study)	88	3	2	5	6	4	6	10	4	16	5	7	4	8	4	2	1	1	–	–	–	–	–	–	–

Keith et al. (2012: table 1) also differentiated the two species based on morphometric characters, such as head and predorsal lengths. However, after examining numerous specimens of both species, it became apparent that these characters also overlapped too much, making them unreliable for differentiation (Table 1). In this study, we identified several morphometric characters that help distinguish *B.segura* from *B.belobranchus*, although the proportional length measurements for these characters overlap between the two species: interorbital width 6.4–11.6% (mean 8.2%) of SL in *B.segura* [vs 4.8–8.0% (mean 6.0%) of SL in *B.belobranchus*]; jaw length 8.7–13.5% (mean 11.1%) of SL [vs 10.3–16.7% (mean 14.1%) of SL]; and caudal-peduncle depth 13.2–16.6% (mean 14.7%) of SL [vs 11.9–15.0% (mean 13.6%) of SL] (Fig. 2).

With respect to coloration, Keith et al. (2012) distinguished *B.segura* from *B.belobranchus* by the upper first dorsal fin being yellowish to orange and the middle and lower parts mottled greyish (vs first dorsal fin uniformly dark brown in *B.belobranchus*). Although our examination of multiple *B.segura* specimens indicates that this character seemed to be consistent, the first dorsal fin of *B.belobranchus* specimens was not always uniformly dark brown, and a thin yellowish hue was observed on the upper first dorsal fin of many individuals (Fig. 3). Keith et al. (2012) also distinguished *B.segura* from *B.belobranchus* based on the color of the second dorsal, anal, and pectoral fins, described as yellowish to orange in *B.segura*. However, our examination indicated that these characters are not consistent (Fig. 3). Moreover, Keith et al. (2012) mentioned one character that seemed relatively consistent for distinguishing between the two species: the caudal fin of *B.segura* was described as never having spots, while the caudal fin of *B.belobranchus* always had spots. Our examination also revealed significant variability in this character, as four of nine *B.segura* paratypes, and many non-type specimens of the species we examined had clearly visible spots on the caudal fin (Fig. 4). However, among the many coloration characters given by Keith et al. (2012) to distinguish these two species, two appear to be reliable: the fresh coloration of the first dorsal fin and the lateral surface of the body (Fig. 3). In *B.belobranchus*, the first dorsal fin is typically uniformly mottled dark brown or exhibits a narrow yellowish distal edge, while the lateral surface of body with many dark horizontal lines (one per scale row). In contrast, *B.segura* consistently has a yellowish to orange upper margin on the first dorsal fin, and the body lacks dark horizontal lines.

The fresh and preserved coloration of the two species, including lines, dots, and bands on the lateral surfaces of the head and body, greatly aid in their identification. For example, *B.segura* has three or four oblique brown lines extending from the eye to the posterior edge of the operculum, compared to one or two in *B.belobranchus*. Additionally, the dorsal and lateral surfaces of the head in *B.segura* lack small whitish spots, whereas *B.belobranchus* has many small whitish spots in these areas. The number of dark-brown bands on the body also differs, with *B.segura* having four or five indistinct bands, compared to 3 distinct bands in *B.belobranchus* (Figs 3–5).

In terms of morphology, *B.segura* exhibits a somewhat different head shape compared to *B.belobranchus*. The dorsal profile of the head in *B.segura* is relatively convex, whereas in *B.belobranchus* the dorsal profile is flatter. In addition, *B.segura* apparently attains a smaller adult maximum size than *B.belobranchus*, the maximum recorded length being 79.8 mm SL (vs 130.7 mm SL in the latter species).

*Belobranchusbelobranchus* and *B.segura* both have distributions ranging from western Indonesia to Vanuatu and as far north as Japan (see Distribution and habitat). The *B.belobranchus* specimens previously reported by Kottelat et al. (1993: pl. 62), Suzuki et al. (1995: fig. 6), Keith et al. (2012: fig. 3B), and Vedra et al. (2013: fig. 2A) from Indonesia, Japan, Vanuatu, and the Philippines, respectively, were identified in this study as *B.segura*, having four oblique brown lines extending from the eye to the posterior edge of the operculum and the absence of small whitish spots on the dorsal and lateral surfaces of the head.

## Supplementary Material

XML Treatment for Belobranchus belobranchus

XML Treatment for Belobranchus segura

## ﻿Additional information

### Author ORCIDs

Muhammad Afrisal https://orcid.org/0000-0002-4809-999X

Nurjirana Nurjirana https://orcid.org/0000-0003-4801-1481

Haryono Haryono https://orcid.org/0000-0002-5143-6790

Daniel Frikli Mokodongan https://orcid.org/0000-0003-4052-0783

Kunto Wibowo https://orcid.org/0000-0003-4465-3022

Abigail Mary Moore https://orcid.org/0000-0002-4122-3740

Nicolas Hubert https://orcid.org/0000-0001-9248-3377

### Data availability

All of the data that support the findings of this study are available in the main text.
